# Conversion of Apricot Cyanogenic Glycosides to Thiocyanate by Liver and Colon Enzymes

**DOI:** 10.5487/TR.2009.25.1.023

**Published:** 2009-03-01

**Authors:** Jiyeon Lee, Hoonjeong Kwon

**Affiliations:** Department of Food and Nutrition, Research Institute of Human Ecology, Seoul National University, Seoul, 151-742 Korea

**Keywords:** Apricot kernel, Rhodanese, Liver, Colon, Subcellular fractions

## Abstract

Some of the edible plants like apricot kernel, flaxseed, and cassava generate hydrogen cyanide (HCN) when cyanogenic glycosides are hydrolyzed. Rhodanese (thiosulfate: cyanide sulfurtransferases of TSTs; EC: 2.8.1.1) is a sulfide-detoxifying enzymes that converts cyanides into thiocyanate and sulfite. This enzyme exists in a liver and kidneys in abundance. The present study is to evaluate the conversion of apricot cyanogenic glycosides into thiocyanate by human hepatic (HepG2) and colonal (HT-29) cells, and the induction of the enzymes in the rat. The effects of short term exposure of amygdalin to rats have also been investigated. Cytosolic, mitochondrial, and microsomal fractions from HepG2 and HT-29 cells and normal male Spraque-Dawley rats were used. When apricot kernel extract was used as substrate, the rhodanese activity in liver cells was higher than the activity in colon cells, both from established human cell line or animal tissue. The cytosolic fractions showed the highest rhodanese activity in all of the cells, exhibiting two to three times that of microsomal fractions. Moreover, the cell homogenates could metabolize apricot extract to thiocyanate suggesting cellular hydrolysis of cyanogenic glycoside to cyanide ion, followed by a sulfur transfer to thiocyanate. After the consumption of amygdalin for 14 days, growth of rats began to decrease relative to that of the control group though a significant change in thyroid has not been observed. The resulting data support the conversion to thiocyanate, which relate to the thyroid dysfunction caused by the chronic dietary intake of cyanide. Because Korean eats a lot of Brassicaceae vegetables such as Chinese cabbage and radish, the results of this study might indicate the involvement of rhodanese in prolonged exposure of cyanogenic glycosides.

## INTRODUCTION

Some edible plants, including bitter almond, apricot, and cassava, contain cyanogenic glycosides. When these compounds are hydrolyzed by β-glycosidase found in microbes or plant tissue, cyanide (HCN) is released (Montgomery, 1965; Osuntokun *et al*., 1970). In acute intoxication, cyanide can cause the rapid inhibition of cytochrome oxidase, resulting in an energy deficit in the target tissues. However, when ingested, cyanide activates the detoxification pathway through rhodanese (a thiosulfate: cyanide sulfurtransferase or TST; EC 2.8.1.1). Rhodanese is a sulfide-detoxifying enzyme that converts cyanides into thiocyanate and occurs in abundance in the liver and kidneys (Ogata *et al*., 1990).

If ingested in sublethal quantities, cyanide is detoxified to thiocyanate within the cells of the organism. However, chronic exposure to cyanogenic glycosides can be goitrogenic, because the thiocyanate, the metabolite, competes with iodide in the thyroid gland, inhibiting the synthesis and clearance of thyroid hormones (Dohan *et al*., 2000). The correlation between cyanogenic plant consumption and the induction of goiter has been reported in many different animal species and humans (Ratnakumar *et al*., 1992; Gaitan *et al*., 1994). Cassava was suspected of having goitrogenic properties for the first time in Nigeria in 1966, where iodine deficiency alone could not account for the frequency of goiter (Ekpechi *et al*., 1966). Recently, many studies have shown the risks associated with the toxic effects of prolonged exposure to cyanide. The etiology of degenerative diseases, such as thyroid disorders (Oke, 1984) and spastic paraparesis ("Konzo"), has been associated with high cassava exposure (Tylleskär *et al.*, 1995). Another pathology associated with cyanide is tropical ataxic neuropathy, characterized by optic atrophy, deafness, Parkinson disease, and spinal ataxia.

There are few botanicals consumed in Korea, either as food or herbal medicine, contain cyanogenic glycosides, including flaxseed, apricot kernel, and peach kernel (Cho, 2007) And also, a natural thioglucoside called "glucosinolate" is consumed as a source of goitrogens present in the *Brassicaceae* family, which includes the cabbage and radish. Thus, activity of rhodanese or thiosulfate-cyanide sulfurtransferase adds risks to the widespread exposure of goitrogen. The objective of this study is to evaluate the conversion of apricot cyanogenic glycosides into thiocyanate by human hepatic (HepG2) and colonal (HT-29) cells, and the induction of the enzymes using rats. The effects of short term exposure of amygdalin in rats have also been investigated.

## MATERIALS AND METHODS

***Extraction of apricot kernel.*** Apricot kernels were purchased from Kyungdong Market, Seoul, Korea and stored at room temperature. One gram of the sample was homogenized by blender in 20 ml of 57 mM potassium phosphate buffer, pH 8.6. The homogenate was centrifuged at 1,000 rpm for 30 min at 4°C and the supernatant was used for experiments.

***Cell culture.*** Human hepatoma HepG2 cells from American Type Culture Collection (ATCC, USA) and human colon carcinoma HT29 cells from Korean Cell Line Bank (KCLB, Korea) were cultured in Dulbecco's modified Eagle's medium (DMEM) and Rosewell Park Memorial Institue (RPMI) 1640 medium, respectively, containing 10% fetal bovine serum (FBS), 100 units/ml penicillin in a humidified atmosphere of 5% $CO_2$ at 37°C. All cell culture reagents were obtained from GibcoBRL (Life Technologies, Cergy-Pontoise, France). At the end of the treatment, the harvested cells were lysed by pestle homogenizer (Tissue grinder 1 ml, Chamber Φ11 × L48 mm, Daihan, Korea) with hypotonic lysis buffer, containing 20 mM Tris (pH 7.5), 5 mM $MgCl_2$, 5 mM $CaCl_2$, 1 mM DTT, 1 mM Ethylenediaminetetraacetic acid (EDTA) (pH 8.8) and protease inhibitor cocktail.

***Animals.*** Male Sprague-Dawley (SD) rats (weighing 170 to 190 g; Laboratory Animal Center of Seoul National University, Korea) were fed commercial laboratory chow (solid) and distilled water, ad libitum. Rats were kept in standard conditions of humidity and temperature with at 12 hour light-dark cycle. After a week of acclimatization, rats (7 in each group) were given a dose of 15 or 30 mg/kg body weight (mg/kg) of amygdalin (Sigma Aldrich) dissolved in saline everyday by gavage. Control group was given saline solution. On the 5, 10 and 15 days, the rats were anaesthetized with zoletile 50 (Virbac labratories) and blood was collected by heart puncture. After the blood collection, the animals were subjected to euthanasia and samples of liver, colon and thyroid were collected and weighed. The health of the rats was checked and their body weights were measured daily. The concentrations of thyroidal hormones, triiodothyronine (T3) and thyroxine (T4) were measured in serum by radioimmunoassay using kits Count-a-Count from DPC® (Diagnostic Products Corporation, Los Angeles, USA). The protocol used in this study was approved by the Seoul National University Animal Use Committee and by IRB (Institutional Review Board).

***Subcellular fractions.*** The homogenate was centrifuged at 800 xg for 20 min. Collected supernatants were sedimented at 20,000 xg for 15 min to separate mitochondria. The resulting pellet was lysed with 300 μl hypotonic lysis buffer to prepare mitochondrial fraction. The supernatant was subjected to ultracentrifugation at 100,000 xg for 60 min in order to separate microsomes from the cytosol. The resulting microsomal pellet was solubilized in 300 μl of the buffer. All procedures were carried out at 4°C.

***Rhodanese assay.*** Rhodanese was measured by the modified method of Sorbo (1953). The reaction mixture was constituted of 250 μl of apricot extracts containing approximately 0.25 M cyanide, 500 μl of 0.125 M sodium thiosulfate, 300 μl of 0.2 M buffer and the enzyme fraction. The assay was initiated by an addition of the subcellular preparations containing 100 μg of protein, and incubated for 60 min at room temperature, then stopped by adding 250 μl of 37% formaldehyde. Color was developed by an addition of 1.25 ml of 0.41 M ferric nitrate solution ($Fe(NO_3)_3 \cdot 9H_2O$ in 14% nitric acid solution) to the reaction mixture and measured at 460 nm, following filiration to remove debris (0.45 μm PES syringe filter, Nalgene). Total protein was determined using the Bio-Rad protein assay reagent.

***Statistical analysis.*** The Student's t-test and 'ANOVA' (one-way) were conducted for testing significances between data of control and treated series at different fixation intervals. Treatment means were compared and separated by Duncan's test at $P < 0.05$ (SPSS12.0K).

## RESULTS AND DISCUSSION

***Conversion of apricot cyanogenic glycosides into thiocyanate.*** Human hepatic and colonal cell homogenates were observed to convert apricot extract to thiocyanate, suggesting the cellular hydrolysis of cyanogenic glycosides to the cyanide ion, followed by its metabolism to thiocyanate. Cyanogenic glycoside that enters through the body system transforms into cyanide after being hydrolyzed in the colon and absorbed and transported to the liver. Thus the colon and liver cells are the first two types of cells that the glycosides encounters. The conversion of apricot cyanogenic glycosides into thiocyanate by HepG2 cells was significantly higher than its conversion by HT-29 cells, both in whole cells and in subcellular fractions ($p < 0.001$). The conversion was highest in the cytosol and lowest in microsomes ($p < 0.001$) (Fig. 1). Rat tissue homogenates also showed same tendency, that is, the conversion was markedly higher in the liver tissue than the colon tissue. The level of cytosolic metabolism was significantly higher ($p < 0.001$) than the mitochondrial or microsomal levels, which did not differ from each other significantly (Fig. 2).

In contrast to previous reports (Ogata *et al.*, 1990) which reported the highest rhodanese activity in mitochondrial fractions, the cytosolic fractions showed the highest rate of the conversion: two to three times higher than that of the microsomal fractions. Cytosolic fraction also showed highest activity when potassium cyanate

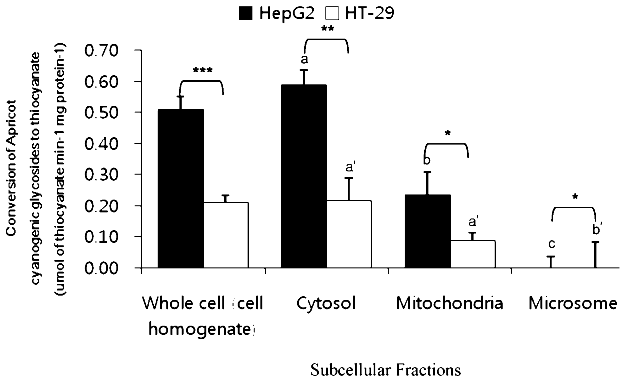

**Fig. 1.** Conversion of Apricot cyanogenic glycosides to thiocyanate by subcellular fractions of established cells. The significant differences between these two cell lines are indicated with asterisks. Indicated different letters (a, b, c; a', b') represent the significant difference in each cell line among subcellular fractions. *: $p < 0.05$; **: $p < 0.01$; ***: $p < 0.001$.

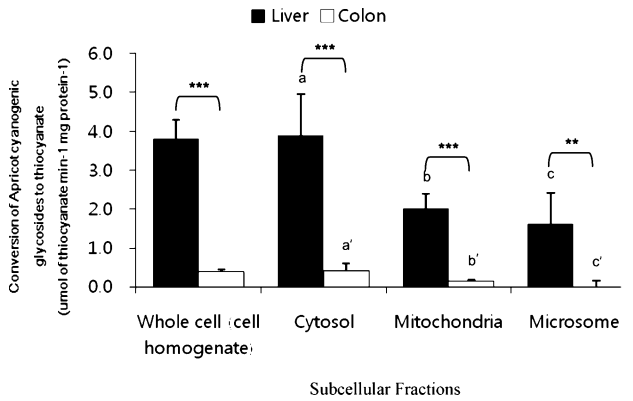

**Fig. 2.** Conversion of Apricot cyanogenic glycosides to thiocyanate by subcellular fractions of tissue cells. The significant differences between these two cell lines are indicated with asterisks. Indicated different letters (a, b, c; a', b', c') represent the significant difference in each cell line among subcellular fractions. *: $p < 0.05$; **: $p < 0.01$; ***: $p < 0.001$.

was used as a substrate. The enzymes known to participate in cyanide detoxification are thiosulfate:cyanide sulfurtransferase (EC 2.8.1.1; rhodanese), 3-mercaptopyruvate:cyanide sulfurtransferase (EC 2.8.1.2; MPST), and cystathionine g-lyase (EC 4.4.1.1.; cystathionase) (Baumeister *et al.*, 1975) but, the assay procedure used in this experiment detects only rhodanese. When cyanide enters the cytoplasm, cytosolic MPST or cystathionase catalyzes a sulfuration reaction. The remaining cyanide enters the organelles enhancing the toxic effects of cyanide, and mitochondrial MPST and rhodanese act together (Porter *et al.*, 1996; Wing *et al.*, 1992).

Unlike other pure chemical cyanides, apricot kernels contain 3% amygdalin, a cyanogenic glycoside, and 50% oil. They also contain an emulsion and a variety of free amino acids. The cytosol of mammalian tissues, such as the liver and kidney, is particularly rich in β-glucosidase activity. All things considered, enzymes in the mammalian cells as well as in the apricot may convert amygdalin to the cyanide ion in the cytosol, followed by its conversion into thiocyanate in the other organelles.

***Rhodanese activity in subcellular fractions of rat liver treated with amygdalin.*** Amygdalin was administered orally to rats to induce rhodanese activity. Rhodanese activities were increased in all the subcellular fractions by the administration of amygdalin to the animal, though statistical significance were only observed in mitochondrial fraction. Still the cytosolic rhodanese activity was markedly higher than that of the mitochondria and microsomes, especially in the liver. A time-dependency of the enzyme induction was also observed

**Table 1.** Induction of Rhodanese activity from subcellular fractions of rat liver and colon treated with amygdalin. Values are mean ± S.D. (n = 7). Values significantly different from control are shown with asterisks ($p < 0.05$ (*))

| | Fractions | Amygdalin (mg/kg/day) | 0 days | 5 days | 10 days | 15 days |
|---|---|---|---|---|---|---|
| Liver | Mitochondria | Control | 7.39 ± 0.71 | 7.51 ± 1.89 | 7.91 ± 2.27 | 7.61 ± 1.53 |
| | | 15 mg/kg | | 7.94 ± 2.37 | 9.42 ± 3.43 | 11.95 ± 3.87* |
| | | 30 mg/kg | | 8.27 ± 2.29 | 10.81 ± 2.32 | 13.15 ± 2.88* |
| | Cytosol | Control | 11.45 ± 0.85 | 11.16 ± 1.60 | 11.28 ± 2.84 | 11.40 ± 1.64 |
| | | 15 mg/kg | | 11.44 ± 2.04 | 13.62 ± 2.59 | 14.39 ± 3.53 |
| | | 30 mg/kg | | 12.28 ± 3.36 | 14.40 ± 1.33 | 14.79 ± 3.92 |
| | Microsome | Control | 5.44 ± 1.33 | 5.33 ± 1.16 | 5.22 ± 1.05 | 5.41 ± 1.42 |
| | | 15 mg/kg | | 5.36 ± 1.53 | 6.05 ± 0.55 | 6.83 ± 1.77 |
| | | 30 mg/kg | | 5.84 ± 1.28 | 6.20 ± 2.06 | 6.88 ± 2.29 |
| Colon | Mitochondria | Control | 2.02 ± 0.13 | 2.01 ± 0.45 | 2.08 ± 0.49 | 2.09 ± 0.73 |
| | | 15 mg/kg | | 2.13 ± 1.22 | 2.29 ± 0.49 | 2.39 ± 0.72 |
| | | 30 mg/kg | | 2.16 ± 0.71 | 2.34 ± 0.57 | 2.49 ± 0.37 |
| | Cytosol | Control | 2.16 ± 0.20 | 2.15 ± 0.38 | 2.16 ± 0.42 | 2.18 ± 0.31 |
| | | 15 mg/kg | | 2.29 ± 0.65 | 2.34 ± 0.41 | 2.50 ± 0.60 |
| | | 30 mg/kg | | 2.38 ± 0.61 | 2.43 ± 0.47 | 2.58 ± 0.47 |
| | Microsome | Control | 1.70 ± 0.10 | 1.69 ± 0.53 | 1.70 ± 0.37 | 1.70 ± 0.29 |
| | | 15 mg/kg | | 1.71 ± 0.17 | 1.71 ± 0.42 | 1.75 ± 0.61 |
| | | 30 mg/kg | | 1.73 ± 0.68 | 1.74 ± 0.22 | 1.77 ± 0.30 |

in the mitochondrial fraction only ($p < 0.05$) (Table 1).

The rhodanese activity in the mitochondria started to increase on the day 15. This finding confirms that long-term exposure to cyanogen increases the enzyme activity of the mitochondria and eventually increases production, which, in turn, suggests that the higher and longer doses of amygdalin probably extend the conversion capacity of the organism. Several studies have suggested that the consumption of cassava increases thiocyanate formation in humans and animals (Ekpechi *et al.*, 1966; Bourdoux *et al.*, 1978; Osuntokun, 1970). Since the demonstration of rhodanese in the liver, which converts cyanide to thiocyanate, it has become clear that this is an effective mechanism for the detoxification of cyanide, but a burden to thoyroid.

***Effect of the amygdalin on rats.*** Liver, colon and thyroid were weighed after the sacrifice at 0, 5, 10 and 15 days of treatments, and the level of blood T3 and T4 were measured at the end of 15 days (Table 2). There were no significant differences in the organ weights (per 100 g body weight), including thyroid, of the experimental group compared with those of the controls at any time during the experimental period though from fourteen days of the treatments, the growth of the treatment group started to decrease (Fig. 3). Neither thyroxine nor triiodothyronine levels were affected in the treated rats (Table 2).

**Table 2.** Weight gain of liver, kidney and thyroid of rats and plasma levels of Triiodothyronine (T3) and Thyroxine (T4) from rats that received different doses of amygdalin during 15 days

| | | | Amygdalin | |
|---|---|---|---|---|
| | Days | Control | 15 mg/kg/day | 30 mg/kg/day |
| Liver weight per 100 g body weight | 5 | 3.13 ± 0.23 | 3.30 ± 0.27 | 3.37 ± 0.12 |
| | 10 | 3.09 ± 0.72 | 3.49 ± 0.37 | 3.26 ± 0.33 |
| | 15 | 3.46 ± 0.05 | 3.54 ± 0.32 | 3.19 ± 0.11 |
| Kidney weight per 100 g body weight | 5 | 0.4 ± 0.03 | 0.39 ± 0.03 | 0.39 ± 0.02 |
| | 10 | 0.4 ± 0.03 | 0.40 ± 0.03 | 0.38 ± 0.03 |
| | 15 | 0.4 ± 0.02 | 0.40 ± 0.02 | 0.36 ± 0.03 |
| Thyroid weight per 100 g body weight | 5 | 0.08 ± 0.04 | 0.07 ± 0.02 | 0.07 ± 0.04 |
| | 10 | 0.07 ± 0.05 | 0.07 ± 0.06 | 0.06 ± 0.04 |
| | 15 | 0.08 ± 0.05 | 0.07 ± 0.03 | 0.05 ± 0.03 |
| T3 (in μg/dl) | 15 | 113.31 ± 6.89 | 112.46 ± 9.54 | 106.26 ± 14.64 |
| T4 (in ng/dl) | 15 | 4.74 ± 0.3 | 4.27 ± 0.82 | 4.74 ± 0.39 |

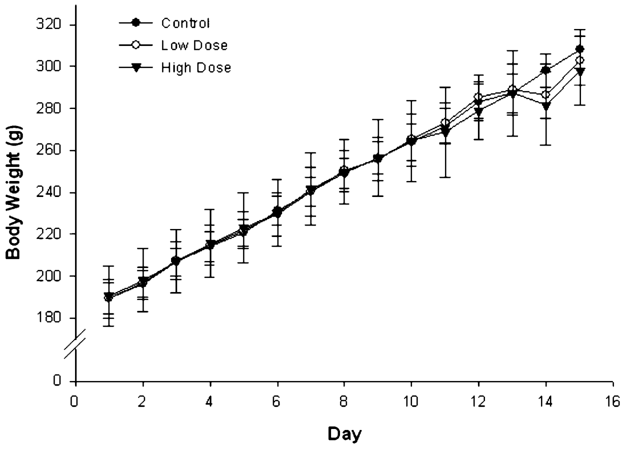

**Fig. 3.** Growth during experimental period. Body weight gains in rats from each experimental group over the entire 15 days. Control: saline-injected rats, Low Dose: 15 mg/kg body weight amygdalin treated rats. High Dose: 30 mg/kg body weight amygdalin treated rats.

Toxic effects of chronic, sublethal doses of cyanide are common in tropical countries, which depend on cassava as a major food source. Some studies have associated weight loss with prolonged CN exposure in humans and several animals, including Wistar rats (De Sousa *et al.*, 2007), sheep (Onwuka *et al.*, 1992), goats (Soto-Blanco *et al.*, 2001), and hamsters (Doherty *et al.*, 1982).

The present study shows that after the consumption of amygdalin for 14 days, growth of rats began to decrease relative to that of the control group. The loss of body weight produced by cyanide appears to result from the depletion of sulfur-containing amino acids from the blood, as they are used as a source of sulfur for cyanide detoxification (Onwuka *et al.*, 1992).The hypothyroidism caused by the excessive consumption of cyanide can also impair the secretion of growth hormones and reduce the number of growth hormone receptors (Lloyd *et al.*, 1990; Koenig *et al.*, 1987). In a study of the guinea pig, Basu's report (Basu, 1983) described the cause of toxicity as the conversion of cyanide into thiocyanate. Another report (Leor *et al.*, 1986) mentioned an amygdalin-administered patient who died from liver damage. It was presumed that the weight of the liver and kidneys would change with exposure to amygdalin. However, the 15 days of exposure in the present experiment showed that the changes of the organ weight were insignificant, raising the possibility that longer exposure is necessary for the toxic effects.

Cyanide affects thyroid function indirectly through its major detoxification product, thiocyanate. The increased levels of thiocyanate that result from subacute cyanide poisoning may be sufficient to markedly depress thyroid function, especially if accompanied by iodine deficiency. In the present study, the thyroid weight had decreased slightly with the amygdalin treatment, although the change was statistically insignificant. Kreutler *et al.* showed that thiocyanate can affect thyroid growth and function in the neonatal rat (Kreutler *et al.*, 1978).

After the consumption of amydalin for 14 days, growth of rats began to decrease relative to that of the control group, though a significant change in thyroid has not been observed. Induction of rhodanese and reduction of weight is not unrelated from the side effects of thiocyanate. Little is known of the induction of rhodanese. Moreover, in case of Koreans, exposure to thiocyanate through *Brassicaceae* family is quite high. Therefore, the prolonged intake of cyanogenic glycosides may increase the thiocyanate burden to thyroids.
